# Supplementary material for: Asynchronous magnetic resonance elastography: Shear wave speed reconstruction using noise correlation of incoherent waves
Source: Magn Reson Med. 2022 Oct 27;89(3):990–1001. doi: 10.1002/mrm.29502 (PMC9792433; doi:10.1002/mrm.29502)
Supplement: Supplementary file 1 — DATA S1 MATLAB code used to generate the numerical simulations of Figure 1 [file MRM-89-990-s002.zip › k-Wave/helpfiles/calculateMassSource.html]

calculateMassSource :: Functions (k-Wave)


# calculateMassSource

Compute k-Wave input plane from measured time-varying data.

## Syntax

```
source_estimate = calculateMassSource(measured_data, dx, dt, c0, source_offset)
source_estimate = calculateMassSource(measured_data, dx, dt, c0, source_offset, grid_expansion)
[source_estimate, output] = calculateMassSource(measured_data, dx, dt, c0, source_offset, grid_expansion)
[source_estimate, output] = calculateMassSource(measured_data, dx, dt, c0, source_offset, grid_expansion, ...)
...
```

## Description

`calculateMassSource` takes a measured 2D plane of time-varying pressure signals (e.g., measured using a hydrophone in a scanning tank) and calculates an equivalent time-varying additive pressure source positioned a given distance away that recreates the measured data when used as an input to k-Wave (i.e., by assigning the equivalent source to `source.p` with `source.p_mode = 'additive'`).

The equivalent source is calculated using an iterative optimisation based on gradient descent, where functional gradients are calculated using the adjoint. Both the forward and adjoint operators are computed using `kspaceFirstOrder3D`. The calculation assumes the propagation is linear and the medium is lossless. The algorithm and approach are described in detail in [1].

If the source is larger than the measured input plane, for example, if measuring a focused bowl transducer, a suitable value should be specified for the value of `grid_expansion`. Note, the value of `source_offset` does not need to match the position of the real source in the experiment.

An alternative approach to project measured data using k-Wave is to directly use the measured data as a pressure source with a Dirichlet boundary condition (i.e., by assigning the measured data to `source.p` and setting `source.p_mode = 'dirichlet'`). However, this approach leads to errors in the imposed spatial gradient, which manifests as errors in the projected field. Thus, for accurate holographic projections using k-Wave, it is recommended to use `calculateMassSource` to first calculate the input data (see [1] for a comparison).

[1] Treeby, B., Lucka, F., Martin, E., & Cox, B. T. (2018). Equivalent-Source Acoustic Holography for Projecting Measured Ultrasound Fields Through Complex Media. IEEE Transactions on Ultrasonics, Ferroelectrics, and Frequency Control, 65(10), 1857-1864.

## Inputs

|  |  |
| --- | --- |
| `measured_data` | 3D matrix containing the time varying pressure over a 2D input plane indexed as (x, y, t) [Pa]. |
| `dx` | Spatial step between grid points in the input plane [m]. |
| `dt` | Temporal step between time points in the input plane [s]. |
| `c0` | Speed of sound in the medium [m/s]. |
| `source_offset` | Offset between the measured input plane and the source plane [grid points]. For example, if `source_offset = 1`, the input plane and source plane are on adjacent grid points. |
| `grid_expansion` | Number of grid points used to expand the size of the estimated source plane in each lateral dimension relative to the measured input plane (set to 0 if not defined). |

## Optional Inputs

Optional 'string', value pairs that may be used to modify the default computational settings.

| Input | Valid Settings | Default | Description |
| --- | --- | --- | --- |
| `'NumSteps'` | *(integer)* | `20` | Number of gradient descent steps. |
| `'StepSize'` | *(numeric scalar)* | `0.5` | Starting size of gradient descent step. |
| `'StepSizeIncrement'` | *(numeric scalar)* | `1.1` | Multiplicative factor used to increase the step size when the error is reduced. |
| `'StepSizeDecrement'` | *(numeric scalar)* | `0.5` | Multiplicative factor used to decrease the step size when the error is increased. |
| `'Plot'` | *(Boolean scalar)* | `true` | Boolean controlling whether the update steps are displayed. |
| `'ReturnIterations'` | *(Boolean scalar)* | `false` | Boolean controlling whether the source estimate at each gradient descent step is returned. If set to `true`, the `source_estimate` output is given as a 4D matrix, each x-y plane corresponds to the source estimate at each step. |
| `'UseCpp'` | `0: MATLAB code` `1: C++ CPU code` `2: C++ GPU code` | `0` | Integer controlling whether the simulations are run using the C++ or CUDA implementations of `kspaceFirstOrder3D`. |

## Outputs

|  |  |
| --- | --- |
| `source_estimate` | If `'ReturnIterations'` is `false` (the default), `source_estimate` is given as a 3D matrix of pressure [Pa] indexed as (x, y, t). If `'ReturnIterations'` is `true`, `source_estimate` is given as a 4D matrix containing the source estimate after each iteration, indexed as (x, y, t, iteration). |
| `output` | Structure containing details of the optimisation with the following fields: |
|  | `.linf_error` |
|  | `.l2_error` |
|  | `.step_size` |
|  | `.number_steps` |
|  | `.number_function_calls` |
|  | `.modelled_data` |

## See Also

`calculateMassSourceCW`, `kspaceFirstOrder3D`
